# Psychological distress and type 2 diabetes mellitus: a 4-year policemen cohort study in China

**DOI:** 10.1136/bmjopen-2016-014235

**Published:** 2017-01-27

**Authors:** C Li, J C Liu, X Xiao, X Chen, S Yue, H Yu, F S Tian, N J Tang

**Affiliations:** 1 Department of Occupational and Environmental Health, School of Public Health, Tianjin Medical University, Tianjin, China; 2 Tongling University, Tongling, Anhui, China; 3 Department of Cardiology, Tianjin 4^th^ Center Hospital, The 4^th^ Center Hospital of Tianjin Medical University, Tianjin, China; 4 Tianjin Emergency Medical Center, Tianjin, China; 5 Medical Center of Police Hospital, Tianjin, China; 6 Tianjin Centers for Disease Control and Prevention, Tianjin, China

**Keywords:** prospective cohort studies, psychological distress, type 2 diabetes mellitus

## Abstract

**Objectives:**

This study investigated whether psychological distress predicts the development of type 2 diabetes mellitus (T2DM) and if the association differs between populations at a high or low diabetes risk level among Chinese police officers.

**Design:**

Prospective cohort study.

**Setting:**

Single centre.

**Participants:**

6559 participants underwent clinical measurements at the hospital in April 2007. 5811 police officers participated in the follow-up consisting of new-onset diabetes (NOD) events occurring annually between 2008 and 2011.

**Primary outcome measures:**

Baseline data were collected from policemen who completed the Symptom Checklist 90-Revised (SCL-90-R) questionnaire and a self-designed questionnaire. Psychological distress was measured by the SCL-90-R questionnaire. Hong Kong Chinese Diabetes Risk Score (HKCDRS) was used to evaluate the risk of T2DM, and the participants were divided into low-risk group and high-risk group based on the HKCDRS. Cox proportional hazards regression was used to calculate the HRs of the incidence of T2DM related to psychological distress and further stratified the analysis based on HKCDRS.

**Results:**

Among 5811 participants, 179 subjects developed NOD during the 4-year follow-up. 54 subjects (1.63%) with a HKCDRS 0–7 vs 125 subjects (4.98%) with a HKCDRS>7 developed NOD (p<0.05). There was a significant association between psychological distress and T2DM (HR=1.46; 95% CI 1.05 to 2.02). Among the participants with a high-risk score (HKCDRS>7), 7.07% of those with psychological distress developed T2DM compared with 4.43% of participants without psychological distress (p<0.05). The corresponding adjusted HR for psychological distress was 1.61 (95% CI 1.10 to 2.37).

**Conclusions:**

Psychological distress is an independent risk factor for T2DM in this prospective cohort study. Stratification analysis indicated that psychological distress was associated with T2DM in a high-risk level population.

**Strengths and limitations of this study**

- This study has evaluated police officers’ physical and mental health conditions by clinical measurement and questionnaire survey in a large size of occupational population samples.
- This study has a high response rate (93%) and few subjects were lost to follow-up.
- This study has examined whether the association between psychological distress and type 2 diabetes mellitus was different in high-risk versus low-risk populations, which have been scarcely studied among Chinese police officers.
- The effects of psychological distress on pre-diabetes were failed to consider.

## Introduction

Type 2 diabetes mellitus (T2DM) is a complex metabolic disease characterised by impaired β-cell dysfunction or insulin resistance.^1^ The hypothalamic–pituitary–adrenal axis and the central sympathetic system are thought to contribute to the metabolic derangement leading to T2DM.^2^ The prevalence of diabetes mellitus is increasing worldwide. The International Diabetes Federation estimated a global prevalence of 382 million people with diabetes in 2013, which is expected to increase to 592 million by 2035.^3^ Compared with developed countries, the incidence of diabetes mellitus is higher in developing countries, India and China have the greatest number of people with diabetes.^4^ As a result of this increasing prevalence, T2DM presents a tremendous clinical and economic burden globally and has become a key public health issue.^5^

Many studies have shown that traditional risk factors such as age, unhealthy habits, obesity, high-fat diet, smoking and excessive alcohol intake are associated with the development of T2DM.^6–8^ However, more recent evidence suggests that psychosocial distress also plays an important role.^9^ ^10^ Previous studies have reported that psychological distress can be a cause of insulin resistance,^11^ and that treatment of psychological factors such as depression can potentially improve glycemic control.^12^ ^13^ Confirmation of this association could have profound implications for the prevention and treatment of these disorders. However, there have been few prospective studies conducted that had evaluated the relationship between psychological factors and T2DM. Furthermore, the findings from these studies are inconsistent.

The association between diabetes and psychological distress is complex, and a simple pattern of cause and effect is not easily discerned. Current studies have focused on the relationship between traditional factors or psychological distress and T2DM. Fewer studies have examined whether these associations with T2DM differ among individuals belonging to a high-risk or low-risk group. The relationship between psychological distress and T2DM has not been well documented among different diabetes risk levels in Chinese policemen, as well. Furthermore, policemen as a special occupational group face more pressure and have a good compliance compared with general population. We chose policemen as our study population to investigate whether psychological factors are associated with chronic disease. Our team has analysed the relationship between psychological distress and dyslipidaemia, clarified the relationship between occupational stressors and the incidence of T2DM based on police officers.^14^ ^15^ In this study, we examined (1) whether psychological distress was an independent risk factor for the development of T2DM in a cohort of Chinese police officers and (2) whether psychological distress at baseline differentially predicted the development of T2DM when data were stratified by T2DM risk levels based on the Hong Kong Chinese Diabetes Risk Score (HKCDRS).^16^

## Study population and method

**Figure 1:**
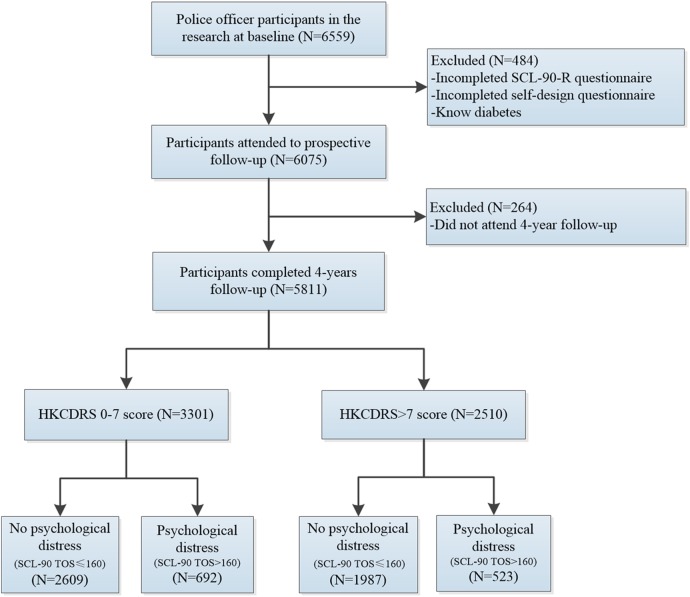
Definition of the study participants. A total of 5811 participants who completed a 4-year follow-up were divided into four groups according to the HKCDRS and psychological distress status. HKCDRS, Hong Kong Chinese Diabetes Risk Score; TOS, total score.

Police officers, aged 20–60 years, were recruited to participate in the current study between 2007 and 2011 in Tianjin, China.^14^ ^15^ A total of 6559 police officers volunteered to participate. Details of the study characteristics are shown in figure 1. At the time of enrolment in 2007, all of the participants had their health status assessed at the Medical Center of Police Hospital and were asked to complete the Symptom Checklist 90-Revised (SCL-90-R) and self-designed questionnaires. The HKCDRS was calculated based on the results of the physical examination and the self-designed questionnaires. Exclusion criteria were the use of insulin or oral hypoglycaemic agents, previously diagnosed diabetes and incomplete information regarding socio-demographic characteristics and mental health status. Finally, the follow-up population consisted of 5811 participants. A clinical examination was performed yearly, and the number of new cases of diabetes between 2008 and 2011 was recorded.

### Ethics

All participants provided informed consent which was provided according to the Declaration of Helsinki.

### Measurements of psychological distress

Psychological distress was assessed by the SCL-90-R, a self-assessment instrument designed to screen for a broad range of psychological disorders.^17^ SCL-90-R includes 90 items that reflect 10 different psychological dimensions: somatisation, obsessive–compulsive, interpersonal sensitivity, depression, anxiety, hostility, phobic anxiety, paranoid ideation, psychoticism and a rest subscale. Each item is rated on a 5-point scale (1–5) of distress. The participant selects one response from the following range of responses: 1=not at all; 2=a little bit; 3=moderately; 4=quite a bit and 5=extremely.^18^ The SCL-90-R was used to clarify specific aspects of a psychological problem and/or psychiatric disturbance.

The Chinese version of SCL-90-R has been used among diverse populations^19^ ^20^ and, at the time of enrolment, was administered to assess the participant's psychological status. The questionnaire's internal consistency was satisfactory with a Cornbrash's α of 0.987 and a Guttmann split-half of 0.961 according to our previous report.^15^ The total score (TOS, range 90–450) was calculated by adding the scores of the subscales. The items relevant to each subscale were means of the total scores for all of the questions related to the symptom. According to the baseline score of the SCL-90-R, participants with TOS that exceeded 160 were classified as mentally unhealthy.^21^

### Hong Kong Chinese Diabetes Risk Score

The diabetes risk score was developed as a feasible way of identifying people at risk of developing diabetes to initiate preventive measures. However, differences in ethnicity and cultural factors called for the development of population-relevant risk scores. In this study, we used the diabetes risk score model based on the Hong Kong Chinese study.^16^ A risk score system was derived using the β values of the corresponding predictors in the logistic regression analysis. The scoring system consisted of five items: hypertension, age, family history of diabetes, body mass index (BMI) and dyslipidaemia. We labelled the model the HKCDRS. The area under the receiver operator characteristic curve was 0.67.

### Measurements of covariates

The survey collected the following information: (1) socio-demographic characteristics including age, educational level, marital status, smoking status, alcohol intake, physical activity level, waist circumference, family history of diabetes and dyslipidaemia. These data were obtained from the self-designed questionnaire; (2) clinical characteristics such as body weight, height, blood pressure, fasting blood glucose and blood lipid levels. Age was divided into three groups (20–39, 40–49 and 50–60 years). A family history of diabetes was defined as having at least one first-degree relative (parents, siblings) or two second-degree relatives (grandparents, uncles or aunts) with diabetes and was categorised as positive or negative.^11^ Smoking habits were categorised as non-smoker, ex-smoker and current smoker, and alcohol consumption was grouped into current drinker, former drinker and never drinker. The BMI was calculated as the weight in kilograms divided by the height in metres squared^22^ and was subsequently divided into three groups (<25, 25–29.99 and ≥30 kg/m^2^). Exercise habits were split into never (less than once per week), occasionally (one or two times per week) and often (more than three times per week).

### Laboratory assay

An overnight fasting venous blood sample was taken between 08:00 and noon and transported to the Medical Center of Police Hospital. Fasting plasma glucose (FPG), blood lipid levels, insulin, serum uric acid, serum urea nitrogen and serum creatinine were measured according to standardised methods. The 75 g oral glucose tolerance test (OGTT) was performed in accordance with the WHO criteria. Blood pressure in the left upper arm was measured three times with a mercury sphygmomanometer after 5 min of rest, and the average was calculated.

### Diagnosis of T2DM

T2DM was diagnosed according to the American Diabetes Association diagnostic criteria when any one of the following symptoms was observed:^23^ (1) FPG ≥7.0 mmol/L (126 mg/dL); (2) an OGTT ≥11.1 mmol/L (200 mg/dL); (3) random blood glucose ≥11.1 mmol/L (200 mg/dL); (4) using insulin or oral hypoglycaemic agents.

### Statistical analyses

The continuous and categorical variables were presented as the mean and SD (mean±SD) and frequencies, respectively. For the comparison of characteristics between participants at baseline with or without the onset of diabetes, we used the χ^2^ test for categorical variables and the independent sample t-test for continuous variables. Comparison of the incidence of new-onset diabetes (NOD) and non-diabetes (ND) was conducted using the Cox proportional hazards regression to calculate the HR and accompanying 95% CI. The HR and 95% CI were computed to compare the effects of traditional risk factors on the incidence of diabetes and were adjusted for covariates including age, smoking status, alcohol intake, education level, physical level, marital status, BMI, family history of diabetes, blood pressure, blood lipids and police classification.

The participants were divided into two groups based on the HKCDRS (score of 0–7, score ≥8). The score of 7 was determined as the cut-off based on the Youden index. Participants were grouped into four groups according to the level of psychological distress (shown in the flow chart, figure 1): (1) HKCDRS 0–7, no psychological distress; (2) HKCDRS 0–7, psychological distress; (3) HKCDRS>7, no psychological distress; (4) HKCDRS>7, psychological distress. The Cox proportional hazards regression was used among the four groups after adjustment for marital status, blood pressure, blood lipids, educational level and police classification.

Statistical analysis was performed using the Statistical Package for the Social Sciences (SPSS) for Windows (V.18.0, Chicago, Illinois, USA). p Values of <0.05 were considered statistically significant, and all p values were two-sided.

## Results

### Population characteristics

Table 1 shows the baseline characteristics of the policemen according to the occurrence of T2DM. Participants with T2DM during follow-up were older, with a greater BMI and a higher SCL-90-R score compared with participants without diabetes. The incidence of hypertension, dyslipidaemia, HKCDRS and police assignment differed significantly between NOD and ND groups (table 1). However, smoking status, alcohol intake, physical activity and a family history of diabetes were not significantly different between the groups.

**Table 1:** Baseline demographic, lifestyle, HKCDRS, working conditions and psychological distress characteristics of subjects according to the occurrence of T2DM

	Non-diabetes (n=5632)	New-onset diabetes (n=179)	χ^2^/t	p Value
Age (years)	37.27±9.01	42.66±9.21	7.764	<0.001
BMI (kg/m^2^)	25.77±3.34	26.97±3.54	4.672	<0.001
SCL-90-R score	134.56±46.80	142.23±50.22		0.031
Marital status (n, %)			0.742	0.690
Married	5214 (92.6)	165 (92.2)		
Other	418 (7.4)	2 (7.8)		
Education level (n, %)			4.396	0.041
College and above	5491 (97.5)	170 (95.0)		
Less than college	141 (2.5)	9 (5.0)		
Exercise activity (n, %)			2.292	0.318
Often (more than three times per week)	1228 (21.8)	31 (17.3)		
Occasion	3747 (66.5)	128 (71.5)		
Never	657 (11.7)	20 (11.2)		
Smoking (n, %)			1.022	0.600
Non-smokers	1808 (32.1)	53 (29.6)		
Current smokers	3218 (57.1)	109 (60.9)		
Ex-smokers	606 (10.8)	17 (9.5)		
Alcohol intake (n, %)			3.348	0.188
Never or almost never	432 (7.7)	13 (7.3)		
Current using alcohol	5068 (90.0)	158 (88.3)		
Former using alcohol	132 (3.3)	8 (4.5)		
Hypertension (n, %)			24.000	<0.001
No	3986 (70.8)	95 (53.1)		
Yes	1646 (29.2)	84 (46.9)		
Dyslipidaemia (n, %)			14.037	<0.001
No	3337 (59.3)	81 (45.3)		
Yes	2295 (40.7)	98 (54.7)		
Family history of diabetes (n, %)			0.157	0.733
No	4109 (73.0)	133 (74.3)		
Yes	1523 (27.0)	46 (25.7)		
HKCDRS (n, %)			65.844	<0.001
0–7 scores	3427 (57.7)	54 (30.2)		
>7 scores	2385 (42.3)	125 (69.8)		
Police assignments (n, %)			97.332	<0.001
Criminal investigation	737 (13.1)	11 (6.1)		
Public security	1484 (26.3)	25 (14.0)		
Administrative services	768 (13.6)	12 (6.7)		
Traffic control	1477 (26.2)	106 (59.2)		
Household registration	799 (14.2)	14 (7.8)		
Other	367 (6.5)	11 (6.1)		

BMI, body mass index; HKCDRS, Hong Kong Chinese Diabetes Risk Score; SCL-90-R, Symptom Checklist 90-Revised; T2DM, type 2 diabetes mellitus.

### Incidence of type 2 diabetes mellitus

**Figure 2:**
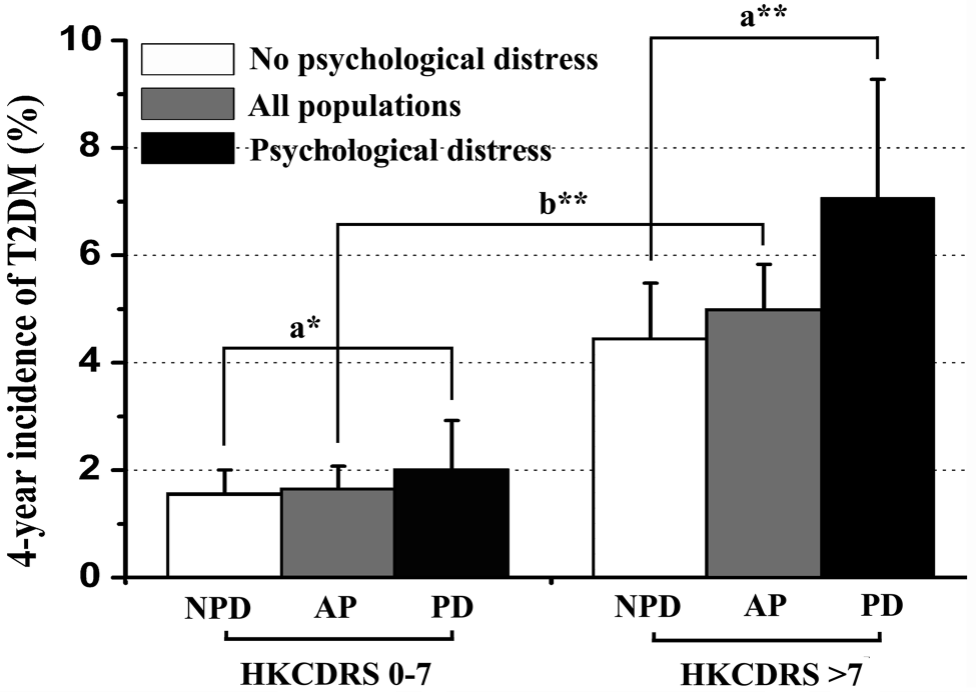
Unadjusted 4-year incidence (95% CI) of T2DM among participants in different HKCDRS groups. The participants were further stratified by the psychological distress status. Unadjusted incidence of participants with psychological distress in HKCDRS of >7 group was higher than participants without psychological distress (p<0.05). AP, all populations; HKCDRS, Hong Kong Chinese Diabetes Risk Score; NPD, no psychological distress; PD, psychological distress. a*: p>0.05; b**: p<0.05; a**: p<0.05.

Among 5811 participants, 179 subjects developed NOD during the 4-year follow-up. The incidence (the number of new cases) of NOD was 34 in 2008, 57 in 2009, 30 in 2010 and 58 in 2011, the annual incidence rate was 9.5/100 person-years. Figure 2 shows the unadjusted incidence, 54 subjects (1.63%) with a HKCDRS 0–7 vs 125 subjects (4.98%) with a HKCDRS>7 developed NOD (p<0.05). Among the participants with a HKCDRS of 0–7, 2.02% of those with psychological distress and 1.53% of those without psychological distress developed T2DM during the follow-up period. The level of psychological distress did not differ in the groups that were at a low risk for the development of T2DM. However, among the participants with HKCDRS>7, 7.03% of those with psychological distress developed T2DM compared with 4.43% of the participants without psychological distress (p<0.05).

### Multivariate analyses of HKCDRS and psychological distress

In the multivariate Cox proportional hazards regression, after adjustments of the covariates, there was a significant association between psychological distress and T2DM (HR=1.46; 95% CI 1.05 to 2.02) (table 2). Results of the multivariable Cox proportional hazards regression confirm a strong association between HKCDRS and the incidence of T2DM. Among subjects without psychological distress, high-risk group (HKCDRS>7) has a higher risk of diabetes compared with low-risk group (HKCDRS<7) (HR=2.66, 95% CI 1.82 to 3.88) in comparison 1 (table 3). Similar results were observed among subjects with psychological distress in comparison 2 (table 3), a significant difference in the association between the HKCDRS and the incidence of T2DM (HR=3.36, 95% CI 1.81 to 6.23). Thus, high diabetes risk score is an independent risk factor for diabetes.

**Table 2:** Cox regression analyses of the association between psychological distress, traditional risk factors and T2DM

	HR	95% CI	p Value
Age (years)			<0.001
<40	Reference		
40–50	1.92	1.35 to 2.75	<0.001
≥50	2.36	1.56 to 3.57	<0.001
BMI (kg/m^2^)			0.132
<25	Reference		
25–30	1.35	0.94 to 1.93	0.102
≥30	1.60	0.98 to 2.63	0.061
Marital status	1.34	0.77 to 2.32	0.294
Education level	1.40	0.71 to 2.75	0.337
Exercise activity			0.184
Often (more than three times per week)	Reference		
Occasion	1.43	0.96 to 2.13	0.079
Never	1.17	0.66 to 2.08	0.585
Smoking			0.653
Non-smokers	Reference		
Current smokers	1.02	0.73 to 1.43	0.889
Ex-smokers	0.80	0.46 to 1.40	0.438
Alcohol intake			0.464
Never or almost never	Reference		
Current using alcohol	1	0.56 to 1.77	0.988
Former using alcohol	1.57	0.64 to 3.84	0.324
Hypertension	1.60	1.17 to 2.20	0.003
Dyslipidaemia	1.41	1.04 to 1.92	0.027
Family history of diabetes	1.04	0.74 to 1.45	0.84
Working hours	1.08	0.80 to 1.47	0.618
Police assignments			<0.001
House registration	Reference		
Public security	0.96	0.50 to 1.85	0.894
Administrative services	0.84	0.39 to 1.85	0.672
Traffic control	3.35	1.89 to 5.93	<0.001
Criminal investigation	0.88	0.40 to 1.95	0.757
Other	1.45	0.65 to 3.21	0.362
Psychological distress	1.46	1.05 to 2.02	0.024

BMI, body mass index; T2DM, type 2 diabetes mellitus.

**Table 3:** Cox regression analyses of the association between psychological distress and T2DM among different HKCDRS groups

HKCDRS and psychological distress at baseline	Sample size	Cases	HR (95% CI) for 4-years incident T2DM			
			Comparison 1	Comparison 2	Comparison 3	Comparison 4
HKCDRS 0–7, no psychological distress	2609	40	Reference	0.78 (0.43 to 1.44)	0.48 (0.27 to 0.85)	0.30 (0.16 to 0.55)
HKCDRS 0–7, psychological distress	692	14	1.28 (0.69 to 2.35)	Reference	0.38 (0.26 to 0.55)	0.62 (0.42 to 0.91)
HKCDRS>7, no psychological distress	1987	88	2.66 (1.82 to 3.88)	2.08 (1.18 to 3.67)	Reference	0.23 (0.15 to 0.37)
HKCDRS>7, psychological distress	523	37	4.29 (2.73 to 6.73)	3.36 (1.81 to 6.23)	1.61 (1.10 to 2.37)	Reference

Alternative reference groups are shown in comparisons 1–4. Models are adjusted for marital status, education level, exercise activity, smoking, alcohol intake, working hours and police assignments. All comparisons are based on the same data, but with a different reference group.

HKCDRS, Hong Kong Chinese Diabetes Risk Score; T2DM, type 2 diabetes mellitus.

We then evaluated whether psychological distress is associated with the incidence of T2DM in two HKCDRS groups. Table 3 shows the association between the combination of the HKCDRS and psychological distress and the risk of T2DM. Comparison 3 indicated that among subjects with a HKCDRS>7, the presence of psychological distress was associated with an increased risk of T2DM (HR=1.61, 95% CI 1.10 to 2.37). However, when compared with subjects with a HKCDRS of 0–7 in comparison 1, with no psychological distress, the HR was 1.28 (95% CI 0.69 to 2.35).

We used a subgroup Cox regression analysis among traffic control police officers to control for the effects of environmental risk factors. Psychological distress in these officers increased their risk of developing T2DM compared with subjects in a high-risk group without psychological distress (HR=1.84, 95% CI 1.06 to 3.17) (table 4). These results were consistent across all populations and indicated that psychological distress predicted the incidence of T2DM in subjects with a high HKCDRS.

**Table 4:** Cox regression analyses of the association between psychological distress and T2DM among different HKCDRS groups in traffic police officers

HKCDRS and psychological distress at baseline	Sample size	Cases	HR (95% CI) for 4-year incidence of T2DM			
			Comparison 1	Comparison 2	Comparison 3	Comparison 4
HKCDRS 0–7, no psychological distress	632	24	Reference	1.75 (0.53 to 5.77)	0.26 (0.08 to 0.83)	0.14 (0.04 to 0.48)
HKCDRS 0–7, psychological distress	174	7	0.57 (0.17 to 1.88)	Reference	0.45 (0.29 to 0.71)	0.54 (0.32 to 0.94)
HKCDRS>7, no psychological distress	620	56	2.20 (1.40 to 3.47)	3.86 (1.21 to 12.35)	Reference	0.25 (0.13 to 0.45)
HKCDRS>7, psychological distress	157	19	4.05 (2.20 to 7.44)	7.09 (2.07 to 24.29)	1.84 (1.06 to 3.17)	Reference

Alternative reference groups are shown in comparisons 1–4. Models are adjusted for marital status, education level, exercise activity, smoking, alcohol intake and working hours. All comparisons are based on the same data, but with a different reference group.

HKCDRS, Hong Kong Chinese Diabetes Risk Score; T2DM, type 2 diabetes mellitus.

## Discussion

In this study, we examined whether the presence of psychological distress at baseline was an independent risk factor for the development of T2DM, and whether the presence of psychological distress differentially predicted the incidence of T2DM at different risk levels according to the HKCDRS. Our main findings were that (1) there was a significant association between psychological distress and T2DM in a multivariate model during a 4-year follow-up of a cohort of Chinese police officers, and (2) psychological distress was associated with a 1.61-fold increase in the risk of T2DM in subjects with a high HKCDRS. These results indicate that psychological distress primarily contributes to the development of T2DM in a high-risk population.

Previous studies have suggested that psychological distress significantly increases the risk of T2DM.^24–26^ In this study, we discovered that psychological distress plays an important role in the development of T2DM. However, initially we found no overall relationship between psychological distress and T2DM in groups with the same HKCDRS. A stratified analysis also revealed no association between psychological distress and T2DM in low-risk subjects compared with participants without psychological distress. The HR was 1.28 (95% CI 0.69 to 2.35) and the difference was not significant. However, in the high HKCDRS subgroup, we observed a 1.61-fold increase in the risk of T2DM in subjects with psychological distress compared with those without psychological distress. In the absence of traditional risk factors, psychological distress may be a primary causative agent in the development of T2DM. Consistent with our results, Marianna *et al* concluded that psychological distress was associated with an accelerated progression to T2DM in individuals with a higher Framingham Offspring Type 2 Diabetes Risk Score.^27^

The debate about the relationship between psychological distress and T2DM is still ongoing. In the Nurses’ Health Study II, occupational distress was not correlated with self-reported T2DM. A series of Whitehall II studies^28^ ^29^ and two studies from Sweden suggested that work-related stress increased the risk of T2DM in women, but not in men.^30^ ^31^ A German study of men working in industry found that psychological distress was associated with diabetes.^32^ ^33^ In addition, Norito Kawakami demonstrated that psychological working conditions were a risk factor for the development of non-insulin-dependent diabetes mellitus in Japanese men.^33^ Current studies have provided confirmatory evidence that psychosocial distress was a potential risk factor for T2DM.^34^ In line with these results, we found a significant relationship between psychological distress and T2DM (HR=1.46; 95% CI 1.05 to 2.02) after multivariate adjustments. The controversy in the literature surrounding the link between psychological stress and diabetes may in part be due to the use of different psychological distress measurement scales and diagnostic criteria for diabetes.

Depression has been shown to be twice as common in people with T1DM and T2DM as in the general population, affecting 10–20% of adults with diabetes.^35^ Researchers suspect that the link between T2DM and depression is in fact a two-way street,^36^ ^37^ and evidence supports the presence of biological correlates of depression in patients with T2DM. A state of high stress may lead to unhealthy lifestyles,^38^ ^39^ such as high-fat diet, smoking, alcohol intake, low physical activity, which may in turn affect HbA1c levels.^40^

In this study, more than 50% of the NOD cases occurred in the group of traffic control police officers (HR=3.35) (tables 1 and 2). The baseline of traditional risk factors for T2DM, BMI, smoking status, alcohol intake, physical activity and other traditional risk factors were not significantly different between the police assignments (see online supplementary table S1) except the constituent ratio of age. Traffic police officers spend a great deal of time working outdoors in China and are constantly exposed to environmental risk factors such as PM_2.5_, automobile exhaust and traffic noise. Studies have shown that environmental risk factors increased the incidence of T2DM.^41–43^ In a pooled meta-analysis, there was a 10% increased risk of T2DM per 10 mg/m^3^ increase in exposure to fine particulate matter (PM_2.5_) and an 8% increased risk of T2DM increased per 10 mg/m^3^ exposure to nitrogen dioxide.^44^ Long-term exposure to total PM increased T2DM risk in the general population, and local traffic-specific PM was related to higher risks of T2DM than the total PM.^45^ However, we have not conducted a panel study to evaluate personal air pollution exposure. In order to certificate psychological distress was associated with T2DM in traffic police officers with high diabetes risk, a subgroup Cox regression analysis among traffic police subjects was conducted. In the subgroup analysis, we observed a 1.84-fold increased risk of T2DM associated with psychological distress in traffic police officers with a high HKCDRS. The external environment, including noise, air pollution, cold and heat, has direct and indirect effects on T2DM. For example, severe conditions in the external environment may contribute to psychological distress. We have previously found that the physical environment functions as an occupational stressor is associated with the development of T2DM.^15^

10.1136/bmjopen-2016-014235.supp1 supplementary table

One plausible mechanism by which psychological distress may predispose to T2DM is through stimulation of the sympathetic nervous system and pituitary gland activity^46^ ^47^ that leads to a rise in circulating catabolic hormones and suppression of anabolic hormone levels.^48^ These neuroendocrine systems are important regulators of glucose uptake, release and storage. The stress response is known to increase cortisol secretion,^49^ which stimulates glucose production in the liver and antagonises the action of insulin in peripheral tissues. This neuroendocrine profile has been associated with abdominal adiposity and increased triglyceride and insulin levels, all of which are established predictors of T2DM. Repeated or continuous activation of these systems during stress, characterised as a paradoxical state of high metabolic activity without corresponding metabolic needs, might predispose to the development of diabetes.

### Limitations

There were several limitations to our study that could influence our results. First, we failed to consider the effects of psychological distress on pre-diabetes, a high-risk condition for the development of diabetes, which can last 10 years.^50^ Second, the SCL-90-R instrument was widely applicated in many studies, but a number of studies have reported the psychometric weaknesses of the SCL-90-R, a central criticism is the postulated factor structure was not replicable in our study. Third, a subgroup analysis indicated that psychological distress increased the incidence of T2DM among traffic police subjects. However, traffic police officers generally work outdoors; we have considered the interaction between external environment and psychological distress, but we have not conducted a panel study to evaluate personal air pollution exposure, which could also potentially affect the progression of diabetes mellitus.

## Conclusion

In conclusion, this observational study found that psychological distress was an independent risk factor for the development of T2DM. The impact of psychological distress on the development of T2DM has healthcare implications that should encourage us to focus on vulnerable populations that are exposed to psychological distress, especially with those belonging to a high-risk group for T2DM.
