# Supplementary material for: Psychological distress and type 2 diabetes mellitus: a 4-year policemen cohort study in China
Source: BMJ Open. 2017 Jan 27;7(1):e014235. doi: 10.1136/bmjopen-2016-014235 (PMC5278237; doi:10.1136/bmjopen-2016-014235)
Supplement: supplementary table [file bmjopen-2016-014235supp_table.pdf]

**Table S1** Baseline of traditional risk factors for T2DM according to the police assignments

| | Criminal investigation (n=748) | Public security (n=1509) | Administrative services (n=780) | Traffic control (n=1583) | Household registration (n=813) | Other (n=378) | $\chi^2$/t | *P* |
|---|---|---|---|---|---|---|---|---|
| Age (n, %) | | | | | | | 429.162 | <0.001 |
| <45 years | 651 (87.0) | 1274 (84.4) | 576 (73.8) | 996 (62.9) | 731 (89.9) | 264 (69.8) | | |
| ⩾45-55 years | 88 (11.8) | 210 (13.9) | 177 (22.7) | 428 (27.0) | 75 (9.2) | 99 (26.2) | | |
| ⩾55-64 years | 9 (1.2) | 25 (1.7) | 27 (3.5) | 159 (10.1) | 7 (0.9) | 15 (4.0) | | |
| BMI (kg/$m^2$) | | | | | | | 18.531 | 0.552 |
| <23 | 167 (22.3) | 302 (20.1) | 172 (22.0) | 298 (18.8) | 166 (20.4) | 74 (19.6) | | |
| ⩾23-25 | 144 (19.3) | 308 (20.4) | 171 (21.9) | 303 (19.2) | 180 (22.2) | 86 (22.8) | | |
| ⩾25-30 | 353 (47.2) | 735 (48.7) | 365 (46.8) | 799 (50.5) | 383 (47.2) | 184 (48.6) | | |
| ⩾30-35 | 80 (10.7) | 156 (10.3) | 66 (8.5) | 170 (10.7) | 80 (9.8) | 33 (8.7) | | |
| >35 | 4 (0.5) | 7 (0.5) | 6 (0.8) | 12 (0.8) | 3 (0.4) | 1 (0.3) | | |
| Marital status (n, %) | | | | | | | | |
| Married | 677 (90.5) | 1394 (92.4) | 740 (94.9) | 1473 (93.1) | 741 (91.1) | 354 (93.7) | 14.284 | 0.014 |
| Other | 71 (9.5) | 115 (7.6) | 40 (5.1) | 110 (6.9) | 72 (8.9) | 24 (6.3) | | |
| Education level (n, %) | | | | | | | 11.138 | 0.049 |
| College and above | 728 (97.3) | 1477 (97.9) | 766 (98.2) | 1526 (96.4) | 797 (98.0) | 367 (97.1) | | |
| Less than college | 20 (2.7) | 32 (2.1) | 14 (1.8) | 57 (3.6) | 16 (2.0) | 11 (2.9) | | |
| Exercise activity (n, %) | | | | | | | 14.284 | 0.014 |
| Often (more than three times per week) | 150 (20.0) | 346 (22.9) | 182 (23.3) | 319 (20.2) | 163 (20.0) | 99 (26.2) | | |
| Occasion | 504 (67.4) | 1028 (68.1) | 520 (66.7) | 104 (65.8) | 545 (67.1) | 235 (62.2) | | |
| Never | 94 (12.6) | 135 (9.0) | 78 (10.0) | 221 (14.0) | 105 (12.9) | 44 (11.6) | | |

| | | | | | | | | |
|---|---|---|---|---|---|---|---|---|
| Smoking (n, %) | | | | | | | 17.217 | 0.072 |
| Nonsmokers | 227 (30.3) | 465 (30.8) | 266 (34.1) | 504 (31.8) | 289 (35.5) | 110 (29.1) | | |
| Current smokers | 443 (59.3) | 881 (58.4) | 420 (53.8) | 911 (57.6) | 455 (56.0) | 217 (57.4) | | |
| Ex-smokers | 78 (10.4) | 163 (10.8) | 94 (12.1) | 168 (10.6) | 69 (8.5) | 51 (13.5) | | |
| Alcohol intake (n, %) | | | | | | | 17.785 | 0.059 |
| Never or almost never | 61 （8.2） | 116 （7.7） | 54 （6.9） | 140 （8.8） | 51 （6.3） | 23 （6.1） | | |
| Current using alcohol | 674 （90.1） | 1352（89.6） | 714 （91.5） | 1394 （88.1） | 748 （92.0） | 344 （91.0） | | |
| Former using alcohol | 13 （1.7） | 41 （2.7） | 12 （1.6） | 49 （3.1） | 14 （1.7） | 11 （2.9） | | |
| Hypertension (n, %) | | | | | | | 14.637 | 0.012 |
| No | 545 （72.9） | 1036 （68.7） | 527 （67.6） | 1134 （71.6） | 591 （72.7） | 248 （65.6） | | |
| Yes | 203 （27.1） | 413 （31.3） | 253 （32.4） | 449 （28.4） | 222 （27.3） | 130 （34.4） | | |
| Dyslipidemia (n, %) | | | | | | | 9.291 | 0.098 |
| No | 428 (57.2) | 925 (61.3) | 451 (57.8) | 942 (59.5) | 450 (55.4) | 222 (58.7) | | |
| Yes | 320 (42.8) | 584 (38.7) | 329 (42.2) | 641 (40.5) | 363 (44.6) | 156 (41.3) | | |
| Family history of diabetes (n, %) | | | | | | | 9.356 | 0.096 |
| No | 533 (71.3) | 1067 (70.7) | 578 (74.1) | 1186 (74.9) | 604 (74.3) | 274 (72.5) | | |
| Yes | 215 (28.7) | 442 (29.3) | 202 (25.9) | 397 (25.1) | 209 (25.7) | 104 (27.5) | | |

Abbreviations: BMI, body mass index;
